# Biocontainment efficacy redefined

**DOI:** 10.1128/aem.01610-25

**Published:** 2025-10-03

**Authors:** Linda F. Bisson

**Affiliations:** 1 Department of Viticulture and Enology, University of California, Davis 117240, Davis, California, USA; University of Illinois Urbana-Champaign, Urbana, Illinois, USA

**Keywords:** *Saccharomyces*, *Brettanomyces*, GMM, biocontainment

## Abstract

Biocontainment of genetically modified microorganisms (GMMs) is thought to be essential to maintain the genetic integrity and lineages of native organisms of the same species in the environment as well as to minimize any ecological risk from release of a novel genetic construct. *Saccharomyces cerevisiae*, a common agent used in food and beverage production, additionally necessitates preventing any risk to human health from accidental penetrance of the GMM in technological sites or lineages. Multiple factors need to be considered in the design of a GMM with the potential for escape to avoid unwanted consequences. Biocontainment efficacy requires understanding the full spectrum of potential ways in which release of a GMM could occur and the impact of that escape from containment on the environment. In an *Applied and Environmental Microbiology* article by N. A. Lamb et al. (91:e00741-25, 2025, https://doi.org/10.1128/aem.00741-25), the authors make the case for a more comprehensive evaluation of biocontainment of GMMs destined for industrial use and raise the critical need of developing and using sound design strategies to assure biocontainment efficacy. The authors demonstrate through analysis of mutants evading genetic biocontainment that existing design strategies have not been adequately evaluated. Although beyond the scope of this research study, sound design strategies should include minimizing the impact to the ecosystem should unintended release of the GMM occur. Potential impact of release requires a deeper understanding of the genomic plasticity of wild and domestic lineages of *S. cerevisiae*. Fortunately, an extensive analysis of the biodiversity of yeast strains isolated from natural and technological ecosystems and their genetic interactions has been conducted and may be useful in redefining concepts of biocontainment efficacy.

## COMMENTARY

Potential negative impacts of unintentional release of a genetically modified microorganism (GMM) can be difficult to predict. Absolute containment with an escape rate of zero may simply be impossible to attain or assure. To increase the efficacy of biocontainment, the development and use of genetic modifications that prevent GMM growth outside of limited permissive conditions has been explored. Permissive conditions selected either do not exist or are unlikely anywhere in the natural environment. Auxotrophies conferring rare nutrient requirements and limiting expression of essential genes have both been used as biocontainment approaches but can negatively impact fitness, drive up costs for supplements and media design, and still allow growth in nutrient-rich environments (1). A combination of growth-limiting strategies can reduce escape occurrence to 10^−10^ (1), well below the suggested National Institutes of Health (NIH) frequency of 10^−8^ (2), but impacts on strain fitness may occur.

An additional approach to biocontainment is the use of “kill switches.” Kill switches are regulatory circuits that when off (or on) lead to the death of the GMM (3). Kill-switch inactive states are maintained under controlled growth conditions in a production facility. Upon release or escape from permissive conditions, the kill switch becomes activated, leading to the death of the organism. This strategy seeks to ensure no impact of the GMM upon release to the environment simply due to nonviability. However, as Lamb et al. (4) point out in a recent article published in *Applied and Environmental Microbiology*, genomes are not static, and through mutagenesis and selection, microorganisms may be able to modify or escape the kill switch, thereby breaching biocontainment. The authors, therefore, evaluated kill switch escape mutants in a model system to define genetic mechanisms that abolish biocontainment. Analysis of the nature of these mutations can then be used to refine design of the GMM for greater biocontainment, or, alternatively, to determine the unworkability of the design strategy.

Lamb et al. (4) elected to evaluate use of a kill switch based on the camphor-regulated RelE toxin system. In this case, the presence of a low level of camphor leads to production of the Cam-transactivator that then represses the expression of the Rel E riboendonuclease toxin. Growth in the absence of camphor leads to loss of repression of the toxin and ultimately cell death. The authors evaluate the impact of this biocontainment strategy on the fitness of the organism as well as assess the ability to escape kill switch containment via mutation. The authors use two yeast strains for this study, a commonly used laboratory strain S288C (5) and derivatives of PE-2, an industrial strain of *Saccharomyces cerevisiae* used in biofuel production in Brazil (6).

Kill switch escape mutational events were evaluated in both the laboratory and technological strain types, as well as with single or multiple copies of the kill switch integrated into the genome. Using single and multiple copies of the kill switch allowed evaluation of mutational events at both primary (components of the kill switch) and secondary (modification of kill switch functionality) sites within the genome. Both primary and secondary site mutations were found in both strains. Not surprisingly, the industrial strain displayed higher escape rates than the laboratory strain. S288C is a domesticated laboratory mosaic strain selected for attenuated plasticity of mutational changes (the hallmark of a good laboratory strain) (5, 7, 8). The industrial strain likely possesses the full suite of components and mechanisms that underpin the well-known ability of *S. cerevisiae* for genome plasticity and adaptive evolution (7, 9, 10).

An extended timeline was used to assess the appearance of escape mutations and growth in the absence of camphor (4). Escape rates were higher in organisms possessing a single copy of the kill switch but also occurred in the presence of multiple copies of the construct. The authors question the validity and practicality of the NIH guideline that suggests safe escape rates should be below 10^−8^ as potentially arbitrary depending upon the time frame selected for appearance of escape mutations to be detected (4). Several of the escape mutations in multicopy strains appeared to be due to the appearance of a mutational event in one copy and then gene conversion (mitotic recombination) to generate a homozygous state for the reversion event and eliminate a deleterious gene. Although mitotic recombination occurs readily in wild populations (11), it remains a relatively rare event.

Opponents of the use of GMMs would likely argue that any potential for biocontainment escape, no matter how low, imposes a risk to the environment. However, an escape rate of “zero” might not be attainable, predictable, or necessary depending upon the genetic modifications being undertaken. For example, a strain (or its DNA) producing a compound considered to be toxic, carcinogenic, or teratogenic would be undesirable were it to escape and mingle with wild or technological populations of *Saccharomyces*, but a strain engineered to produce higher levels of a growth nutrient might not pose a risk.

Tiered frameworks for assessment of risk associated with GMM release based on either the possibility of the genetic change occurring in nature or the relatedness of the primary sequence to the organism being modified. Closeness to self or “natural” being seen as relatively harmless as compared to non-species or fully synthetic DNA (12). Introduction of non-species genetic material from a natural or completely synthetic source would require the highest level of scrutiny. But what truly is outside of the scope of a natural genetic modification occurring in the environment? What is the actual risk to population and genomic integrity of release of a manufactured organism or DNA to natural environments?

*S. cerevisiae* is an ideal organism in which to address these questions as there is a vast amount of published data on genome variation of wild and technological strains and their dispersal by human migration and activity (11, 13–18). These studies reveal an impressive level of genetic and genome diversity not predicted from analysis of laboratory lineages of *S. cerevisiae* (19, 20). In fact, wild and technological strains of *S. cerevisiae* span the full spectrum of the three proposed tiers for biocontainment.

Given the importance of *S. cerevisiae* to numerous food and beverage technologies and the belief that autochthonous diversity underpins regional identity of specific products, genetic diversity of *S. cerevisiae* as a function of geography of origin and site of origin has been assessed (11, 16, 18, 21, 22). Distinct domestic and wild lineages are evident as well as mosaic lineages indicating gene transfer between the two populations (16, 23). Wild lineages are generally specific to geographic regions, while domestic lineages seem to be associated with technological processes regardless of geographic location. Domestication events in food and beverage production occurred in multiple geographic locations roughly 3–7,000 years ago as documented by independent lineages (18, 24). The extent of biodiversity and number of wild lineages identified in China suggest *S. cerevisiae* originated in Asia with the likely niche for *S. cerevisiae* being oak trees and primary forests (18). These studies have revealed geographic lineages, technological lineages across geographic barriers, and technological lineages limited by geography (16). Strains seemingly isolated to the winery and not found in adjacent vineyards also exist (25).

A detailed analysis of the population structure of *S. cerevisiae* in a wine-growing region of Canada (23) identified four distinct lineages or clades: Wine/European, Transpacific Oak, Beer/Mixed Origin, and a new clade designated Pacific West Coast Wine. This fourth clade showed the greatest biodiversity with representation in the lineage from North American oak strains intertwined with gene flow from Wine/European and Ecuadorian clades. Thus, technological lineages display significant diversity and cross flow of genes from and to wild lineages as well as from other technological lineages.

The remarkable diversity of strains across these lineages showcases the extensive genomic plasticity of *S. cerevisiae*. Point mutation, insertion/deletion, chromosomal rearrangement, aneuploidy, polyploidy, gene copy number variation, horizontal gene transfer, outcrossing, mitotic recombination, sexual reproduction, and the formation of viable hybrids across species of *Saccharomyces* provide an extensive tool kit for genomic change (7, 9, 26–30). Stress-induced, spontaneous, and directed mutagenesis also contribute to a high capacity for genome tinkering and subsequent adaptive evolution (31–37).

Yeast strains may be heterothallic, sexual reproduction only via outcrossing across unique meiotic events or mating between haploid spores of the same meiotic event. Strains may also be homothallic with the ability of a single spore to switch mating type, allowing genetically identical (except for expressed mating type) haploid spores to mate, forming a fully homozygous diploid. Homothallism leads to the phenomenon of complete loss of heterozygosity via a process termed “genome renewal” (10). Genome renewal allows the fixation of advantageous and loss of deleterious mutations (10). Lamb et al. (4) suggest as one rule for biocontainment use of heterothallic starting strains unable to engage in genome renewal. However, analysis of vineyard populations of *S. cerevisiae* indicates that outcrossing and mitotic recombination to attain homozygosity is more common than mating type switching and mating of progeny from a single meiotic event (27). In addition, yeast strains display the capacity for heritable phenotype switching that is protein-dependent (38, 39). Phenotypic plasticity extends beyond genotypic.

The initial study of strain diversity by technological origin (24) identified a lineage cluster centered around a single wine strain, UCD522. The UCD522 cluster showed the least geographical isolation and was identified in vineyards on multiple continents. UCD522 is a good case study for biocontainment efficacy. This strain was isolated from a French wine and because of robust fermentation properties in dominating and completing wine fermentations was used for wine production widely in California (40). These traits led to the commercialization and worldwide distribution of UCD522. What made that strain excel at dominating grape juice environments is thought to be the ability to produce high quantities of hydrogen sulfide (40, 41). H_2_S plays an important role in cell signaling via coordination of population metabolism to switch from respiratory to fermentative metabolism (21, 41). It also stimulates proliferation and leads to sustained viability (21). Hydrogen sulfide itself can lead to off-odors in wine and the strain fell out of favor and was replaced by other commercial strains in winery settings. The persistence of UCD522 in vineyards in multiple geographic regions where it was once used as a wine strain indicates that a genotype can serve strains well in a variety of ecosystems and, if so, will mingle with existing lineages and persist even when no longer being continually introduced as a commercially produced inoculum.

Biocontainment efforts for *S. cerevisiae* focus on the design of genomic modifications leading to attenuated strains with a limited permissive growth environment. The inherent assumption that escape risk is exclusively correlated with appearance of reverse or mitigating secondary mutations and that escape will enable genetic exchange with wild lineages is fully validated by the analysis of wild and technological lineages.

In addition to the UCD522 example of single strain survival and ecosystem persistence in diverse geographic regions, there is also evidence of single gene penetrance of lineages. A classic example of human activity selecting for specific genome rearrangements in a technological lineage is the use of sulfur dioxide as an antimicrobial agent in wine making (21). Yeast strains contain a sulfur dioxide pump, the *SSU1* gene, conferring resistance to this compound toxic to many other microbes in grape environment. Genome rearrangement events in the promoter region of this gene between non-homologous chromosomes (42–44) led to the formation of dominant alleles overexpressing the *SSU1-R* gene conferring greater resistance to sulfur dioxide. Increases in copy number (from two to six copies) of the rearranged dominant allele *SSU1-R* have also been found (42, 43). Roughly 50% of the wine isolates examined carry this specific chromosomal rearrangement (43). The widespread occurrence in nature of this specific chromosomal rearrangement suggests that the pervasive use of sulfite by humans led to a growth advantage to strains carrying a *SSU1-R* and that the allele became fixed in lineages. Thus, both strains and genes that confer an environmental advantage can become fixed in geographic and technological lineages. Sound design strategies for GMMs should use constructs and genetic backgrounds not conferring a growth advantage in wild or technological ecosystems.

Analysis of wild and technological lineages indicates natural hybrid formation between species of *Saccharomyces* is common across geographic ecosystems. Hybrids of *S. cerevisiae* and *S. kudriavzevii*, *S. bayanus*, and *S. pastorianus,* as well as triple hybrids of *S. cerevisiae, S. kudriavzevii,* and *S. bayanus,* have been identified multiple times in wine and vineyard populations (45–48). Hybridization occurred between haploid or diploid cells. Although members of the same genus, these species are isolated with respect to the inability of mating of haploid progeny. Following cross-species hybridization, chromosomal loss, rearrangements, and chimeric chromosome formation may occur (45). Natural selection then enriches for the most-fit outcomes of hybridization. The surviving populations of these events are stable mitotically but may or may not be able to undergo meiotic division and engage in sexual reproduction or outcross to one of the parent species of the hybridization event (21). If outcrossing occurs, non-species DNA may be spread to genetic backgrounds that did not participate in the original hybridization event. Thus, *S. cerevisiae* is not genetically isolated from other members of the species, which should also be considered in GMM design.

In wine strains of *Saccharomyces*, the evidence of ancient hybridizations is also detectable as introgression of DNA segments from unrelated organisms (49). It is not clear if introgression arose due to large region horizontal gene transfer events, the pickup of DNA from the environment, or abortive hybrid formation between unrelated yeasts (21). Wild and domestic lineages of *Saccharomyces* show the introduction of foreign DNA from other organisms appearing as lateral or horizontal gene transfer. Genome tinkering in *S. cerevisiae* is not limited to self or related species sources.

A case in point is wine strain EC1118. Analysis of the EC1118 genome revealed three independent introgression events in the history of the strain. These introgressions carried a total of 34 functional genes and resulted in the expression of unique phenotypic traits (14, 49). One introgression arose from a member of the same genus, *S. pastorianus*, a second region arose from *Zygosaccharomyces bailii,* a yeast also found in similar environments, and the third region was fungal in nature but from an undetermined source (49). The introgression from *S. pastorianus* contained an analog of the *FSY1* gene encoding a fructose proton symporter (50). If functional as such in EC118, this transporter would couple fructose movements to proton uptake, resulting in more efficient transport of fructose in the low pH environments common in grape juice. The unknown fungal donor introgression encodes a protein similar to peptide transporters found in fungi but absent in *S. cerevisiae*, thereby expanding the array of peptides that can be used as nitrogen source (51). The introgression of the presumptive *Z. bailii* donor (49) is not unique to EC1118 and is found in other wine yeast strains (14, 50). This region contains an autonomously replicating sequence, suggesting that it may be able to amplify itself and adjacent genes in the genome leading to copy number variation. In support of the amplification hypothesis, some wine strains have been found to carry multiple regions of this DNA (50). The functions of this region are unknown, but its appearance in many wine strains suggests an advantageous role. That a single wine strain carries multiple advantageous genes derived from multiple introgression events suggests that uptake and genomic incorporation of large regions of external DNA may readily occur in *S. cerevisiae*. Sound GMM design should consider the possibility that recombinant DNA may be transmitted to ecosystem members in the absence of cell fusion or sexual reproduction.

The observation of previously thought rare cross-species hybridizations and the appearance and stabilization of foreign DNA introgressions raised the obvious question of the mechanism of occurrence in native environments. Potential clues to the niche in which such genomic exchange could occur came from the process of digestion of yeast tetrad walls for genetic analysis (52) and the development of a protoplast fusion technique under laboratory conditions for the artificial creation of hybrid strains (53). Both tetrad dissection and protoplast fusion require treatment of cells with cell wall digestive enzymes derived from the snail gut (52, 53). The usefulness of insect enzyme preparations in controlled yeast tetrad and vegetative cell wall digestion led to investigation of the role of insects in both hybrid formation and genetic plasticity in *Saccharomyces*.

Fruit flies from the genus *Drosophila* are often found in the same fermentative environments as *S. cerevisiae*. Viable yeast haploid spores were found in the feces of *D. melanogaster* feeding on *S. cerevisiae* (54). Viable vegetative cells were not found, suggesting spore walls are able to survive exposure to digestive enzymes, be deposited in feces, enabling diploidization with spores of the opposite mating type. Feeding two genetically distinct strains of *S. cerevisiae* to flies resulted in tenfold higher recovery from feces of outcrossed strains compared to controls of mixed cultures in the absence of fly feeding. Thus, fruit flies can act as both dispersal agents of yeast and promoters of sexual outcrossing in the ecosystem.

Subsequent work demonstrated that fungal ascospores in general survive passage through the fruit fly gut while vegetative cells do not, suggesting that ascospores may have evolved specifically for insect dispersal (55, 56). Other insects have been identified as facilitating not only sexual reproduction in *S. cerevisiae* but also cross-species hybrid formation and horizontal gene transfer (57–59). Insects, therefore, play a vital role in the generation of biodiversity in yeast natural environments and in the transmission of yeast within and across those ecosystems. Interestingly, attraction of fruit flies to yeast fermentations has been shown to be due to aromatic metabolites of the yeast and not of the fruit itself (60), suggesting a strong mutualistic relationship between insect and yeast. Yeast would provide nutritional content to the fly from feeding on vegetative cells in exchange for the fly providing a niche for sexual reproduction and cross-species genetic exchange and a mechanism of spread. Sound biocontainment strategies would also need to consider the potential role of insect vectors in facilitation of GMM dispersion and genetic exchange.

The genomic plasticity and genomic and genetic biodiversity of wild and technological lineages of *S. cerevisiae* are astounding. Given the rich genomic complexity and variability of *S. cerevisiae*, the creation of GMM organisms for wine production has largely been abandoned in favor of mining existing biodiversity (61). Exchange of DNA is not limited to within the species or genus. The mutualism of insect feeding, genetic exchange, and species dispersal all but assures any novel introduced genome will be fully vetted for advantageous genes which can then be fixed in wild or technological lineages. The introduction of a GMM into any of these ecosystems may add to this diversity but is unlikely to disrupt it. The risk of release of a GMM reduces to risk to the environment of transmission of the modified DNA to wild and technological strain. Use of attenuated strains less likely to survive in the natural or technological environments and unable to exchange DNA with organisms within these ecosystems becomes tantamount to sound GMM design.

The Brazilian biofuel strain used in the analysis of kill switch efficacy was originally identified as a robust strain for biofuel production (6). Although a good candidate for bioproduction processes, especially those using readily available substrates, the higher rates of gene conversion observed (4) might not be desirable in a GMM. Further, biofuel fermentations are often overrun with contaminating organisms (62, 63), one of which is the yeast *Brettanomyces* often found in similar technological environments as *S. cerevisiae*. Bacteria and other yeast strains also present as contaminants (62). A strain of *S. cerevisiae* able to dominate such mixed competitive populations might contain environmentally advantageous phenotypes in its genome that would render it undesirable as a genetic background for a GMM.

The dominance of *Brettanomyces* in bioethanol production facilities led to the suggestion that this yeast be used as an alternative to *S. cerevisiae* in biotechnological processes (63–66). Given its much broader substrate range and heightened competitiveness or coexistence with organisms inhibiting *S. cerevisiae,* the lower yield in ethanol from hexoses may be overcome by the ability to ferment a wider range of carbon sources present (64, 67). *Brettanomyces* is best known as a spoilage contaminant of biotechnological processes leading to deterioration of sensory quality (68). It is associated with wine and beer spoilage, although niche markets exist for lambic beers and “Bretty” wines that rely on the peculiar aroma and flavor modifying traits of this yeast. *Brettanomyces* is rarely found in natural environments outside of a production facility (68) but can be isolated from vineyards with enrichment (69, 70). Multiple strains of *Brettanomyces* have been sequenced, and the genomic plasticity extends well beyond what is seen for *Saccharomyces* (68, 71).

Members of *Brettanomyces* can be found in diploid or triploid (two homologous chromosomes plus an unrelated haploid genome) states (68). The triploid state precludes sexual reproduction but facilitates genetic exchange via mitotic recombination. Striking karyotype variability also exists in *Brettanomyces* with analyzed isolates showing between 4 and 9 chromosomes (72). The presence of large regions of homology in *Brettanomyces* in the absence of genome renewal suggests mitotic recombination is amplified in this organism (71). Thus, many of these strains are isolated genetically from other members of the species given differing karyotype and ploidy.

*Brettanomyces* shows extensive adaptive mutation ability, often becoming terminally adapted to the facility and not found elsewhere in the environment. It has been suggested that *Brettanomyces* may be better for the design of GMMs with novel properties given its predilection to adapt to a facility at the expense of return to a more natural environment (63, 64, 66, 67). Use of a triploid genome would prevent any genetic exchange with the more common diploid genomes of wild species. Sound GMM design strategies may have to look beyond the genus *Saccharomyces*.

### Conclusion

Lamb et al. (4) make a cogent case for identifying design rules facilitating biocontainment efficacy. But what should those rules be given the extensive genome tinkering toolkit possessed by wild and domestic lineages of *S. cerevisiae*? The realities of biocontainment efficacy should be considered in any evaluation of risk of release. Rather than thinking in terms of an acceptable escape frequency, escape should perhaps be assumed. Background strain selection should assess potential advantages of transfer of the GMM or its DNA to wild or technological populations. Strains should be developed to be defective in sexual reproduction and ability to attract insects. As proposed by Lamb et al. (4), genes underpinning adaptive evolution and mitotic recombination should also be inactivated. The ability of the starting organism to persist in technological environments should be assessed and non-competitiveness in any technological process producing food or beverages selected. Most importantly, risk can be reduced by choosing a GMM host that is not as widespread across wild and technological ecosystems as is *S. cerevisiae*. The concept of biocontainment efficacy may need to be expanded beyond containment in a facility to natural biocontainment if released.
